# Recent developments in bibliotherapy for adolescent depression

**DOI:** 10.3389/fpsyt.2025.1681462

**Published:** 2025-12-12

**Authors:** Fei Liao, Jiaquan Liang, Meng Zhang, Wenting Liang, Chunguo Zhang, Xuan Yun

**Affiliations:** 1 Department of Psychiatry, The Third People’s Hospital of Foshan, Foshan, Guangdong, China; 2 The Foshan Library, Foshan, Guangdong, China

**Keywords:** bibliotherapy, adolescent depression, advances in research, mechanisms of action, intervention approaches

## Abstract

**Objective:**

This review systematically examines the current research on bibliotherapy for adolescent depressive disorders, emphasizing its efficacy and potential as a non-pharmacological intervention, while also considering the variations across different types of bibliotherapy and inherent limitations of the intervention itself.

**Methods:**

A narrative review of the literature was conducted to explore the therapeutic mechanisms of bibliotherapy—including emotional resonance, cognitive restructuring, and social support—and to analyze various application models, such as guided versus unguided formats, creative versus self-help bibliotherapy, group reading, individualized interventions, and emerging digital approaches.

**Results:**

Evidence indicates that bibliotherapy can effectively reduce depressive symptoms in adolescents across diverse intervention strategies. Both creative and self-help bibliotherapy, as well as group-based and individualized formats, have shown promising outcomes, particularly when integrated with structured discussion or other therapeutic approaches like CBT and mindfulness. However, the intervention’s effectiveness can be moderated by factors such as text selection, reader characteristics, and cultural context. Potential limitations include emotional triggering from certain narratives, variability in individual engagement, and inconsistent long-term efficacy, especially among adolescents. Existing studies are further constrained by small sample sizes, brief intervention periods, and a lack of standardized material selection criteria.

**Conclusion:**

Bibliotherapy represents a valuable, accessible intervention for adolescent depression, yet its application requires careful implementation to mitigate potential drawbacks. Future research should focus on large-scale, multicenter trials, further investigation of neuroscientific mechanisms, development of personalized reading programs, establishment of practitioner training systems, and cross-cultural adaptation. Efforts should also address ethical and privacy concerns, particularly within digital platforms, and explore creative integration with complementary therapeutic methods to enhance overall efficacy and safety.

## 1 Introduction

Depression is one of the most common psychological disorders among adolescents, with its prevalence increasing more rapidly in this group than in adults (1). In China, the growing concern over adolescent mental health has prompted the Ministry of Education to implement nationwide mental health screenings, highlighting the emphasis on early identification and intervention (2). Adolescent depression is characterized by chronicity and high recurrence, often accompanied by severe functional impairment and increased risks of self-harm and suicide (3, 4). Common consequences include interpersonal difficulties, academic decline, and sleep or cognitive disturbances (5–7).

Pharmacological treatments for adolescent depression are often limited by unpredictable side effects and developmental vulnerability, creating a need for safer, more accessible non-pharmacological interventions. While techniques such as transcranial magnetic stimulation and art or music therapy has shown promise, many require specialized equipment or settings (8–11). In this context, bibliotherapy—a low-cost, flexible intervention using literature for emotional and psychological healing—offers distinct advantages. It facilitates self-reflection, emotional resonance, and cognitive restructuring, and can be applied outside clinical environments (12, 13).

As a non-pharmacological intervention, bibliotherapy has gained increasing attention in the field of adolescent mental health in recent years. This article systematically reviews the research progress of bibliotherapy in the treatment of adolescent depression, offering insights to guide clinical practice and future studies.

## 2 Definition and classification of bibliotherapy

The concept of bibliotherapy can be traced from ancient times to the modern era (14–16). As technology has advanced, alternative forms of media have been adopted. Bibliotherapy is defined as the use of written materials, computer programs, or audio/video resources to facilitate understanding or address challenges related to personal development and therapeutic needs (17). It operates in a way similar to therapist-led interventions, as books or manuals incorporate psychotherapeutic principles aimed at increasing individuals’ awareness of negative emotions, providing effective coping strategies, and encouraging the practical application of these methods in daily life (18, 19). Initially, bibliotherapy was developed as an intervention for depression (20). Bibliotherapy began to gain formal recognition in the early 20th century and was widely incorporated into mental health services in psychiatric hospitals and school-based interventions, especially after World War II.

According to the method of intervention, bibliotherapy can be broadly categorized into two types: guided bibliotherapy and unguided bibliotherapy. Guided bibliotherapy is typically facilitated by a mental health professional and includes structured components such as recommended reading materials, reading guidance, emotional expression, cognitive discussions, and behavioral suggestions. It emphasizes systematization and structured engagement (21). In contrast, unguided bibliotherapy allows individuals to choose and read independently, fostering self-reflection. Although the therapeutic outcomes are harder to control, this approach is more accessible, cost-effective, and easier to disseminate, making it suitable for individuals with higher cognitive ability and intrinsic motivation.

Furthermore, bibliotherapy encompasses a continuum ranging from the use of literature to promote health and well-being to its application as a form of clinical psychological intervention (22). Brewster divides bibliotherapy into two main models: self-help bibliotherapy and creative bibliotherapy (23). The former utilizes non-fiction self-help books recommended by medical professionals to provide practical support for individuals with mental health issues, while the latter employs novels, poetry, and other literary works to engage with individuals or groups, fostering mental well-being and emotional growth (24). Research has well established that self-help bibliotherapy produces significant effects on various mental health and well-being outcomes across different age groups (25, 26). However, the evidence base for creative bibliotherapy remains relatively limited. Troscianko further pointed out that current research and practice in creative bibliotherapy are still insufficient in understanding its mechanisms of action (27).

In recent years, the rise of digital technology has led to the emergence of e-bibliotherapy, which leverages e-books, mobile applications, and online reading platforms to enhance accessibility and interactivity. This digital approach is particularly appealing to adolescents who are digital natives (28).

## 3 Theoretical foundations of bibliotherapy- psychological mechanisms

Bibliotherapy has been widely applied in the treatment of adolescent depression, particularly in the area of cognitive restructuring. Cognitive restructuring refers to the process of improving emotional states by changing individuals’ interpretations of specific events or situations. A large body of research has shown that bibliotherapy can effectively alleviate depressive symptoms, for example, through reading relevant books or manuals such as Feeling Good, Mind Over Mood, The Good Mood Guide, and Doing What Matters in Times of Stress: An Illustrated Guide (29). Notably, the cognitively focused Feeling Good (30) and the behaviorally oriented Control Your Depression (31) are among the most widely utilized examples (32). Both clinical and statistical evidence have demonstrated its therapeutic efficacy in alleviating depression (33). Studies have shown that the core mechanism of bibliotherapy may involves guiding individuals to read texts that align with their psychological state, thereby eliciting emotional resonance, promoting self-reflection, and facilitating cognitive restructuring to support emotional regulation and psychological recovery (34).

Through carefully selected literary works, adolescents are exposed to a range of emotional experiences and values, enabling cognitive reshaping. For example, the challenges and triumphs experienced by characters in certain stories can prompt adolescents to reflect on their own situations, leading to a shift in their negative perceptions of themselves and the world around them. Literature has shown that reading can help adolescents identify and challenge deeply held negative beliefs, thus potentially contributing to the alleviation of depressive symptoms (35). Moreover, reading allows adolescents to experience others’ emotions and struggles in a safe environment, and this development of empathy is also a key component of cognitive restructuring.

Creative literary works possess the capacity to evoke profound emotional engagement, guiding readers into a narrative realm that is both aesthetically pleasing and psychologically enlightening. Certain works offer a psychologically safe space for emotional exploration, allowing readers to reflect upon and regulate their own affective experiences through empathy with the characters (36). Research indicates that the cognitive processing of fictional and non-fictional texts differs significantly, with distinct neural networks being activated in each case (37). Moreover, fictional narratives may enhance individuals’ capacity for perspective-taking, a phenomenon closely linked to the brain’s mechanisms for constructing and comprehending narrative contexts (38). This effect, known as the “transportation effect”, describes the ability of stories to temporarily disengage readers from the real world and immerse them within the narrative universe. Scholars suggest that this form of transportation “may lead to real-world belief (and behavior) change” (39). When readers share demographic or experiential similarities with the characters—such as age, gender, or life history—the depth of psychological immersion tends to increase (40). Moreover, engaging in shared reading experiences with others (as in group bibliotherapy) facilitates emotional connectedness and social support, both of which are regarded as fundamental components in the recovery of mental well-being (41).Such texts not only offer a channel for emotional catharsis but also provide strategies for coping with personal emotions.

Through the process of reading, adolescents can establish connections with characters in the texts, which fosters the development of self-empathy and a sense of identification. This identification can enhance adolescents’ sense of social connectedness and help reduce the occurrence of depressive symptoms. Research has found that when adolescents encounter storylines that mirror their own experiences, they may feel understood and accepted, which could significantly eases their feelings of loneliness (42). In this way, reading not only provides emotional support but also promotes self-understanding and self-acceptance. Additionally, reading helps adolescents explore their identity, comprehend their emotions and experiences, and thereby enhance their self-efficacy and confidence. The development of self-identity plays a crucial role in reducing depressive symptoms and enhancing psychological resilience (43, 44). Therefore, as a form of psychological intervention, bibliotherapy can play an important role in the treatment of adolescent depression by fostering self-empathy and a strong sense of identification.

## 4 Application of bibliotherapy in adolescent depression

In recent years, there has been a growing body of international research on the application of bibliotherapy in the treatment of adolescent depression. These studies primarily focus on evaluating its therapeutic efficacy, exploring innovative intervention models, and examining its integration with other psychological treatment modalities. Collectively, they provide an emerging evidence base for the development of bibliotherapy in this population.

### 4.1 Intervention effectiveness of bibliotherapy

Research indicates that bibliotherapy may have a certain effect in alleviating depressive symptoms in adolescents. A meta-analysis by Yuan et al. (44) synthesized multiple randomized controlled trials targeting depression and anxiety in children and adolescents, reporting a standardized mean difference (SMD) of –0.52 (95% CI: –0.89 to –0.15), suggesting a moderate intervention effect. The analysis also found that participants receiving bibliotherapy appeared to showed significant reductions in depression scores, with effects maintained during follow-up. Additionally, the therapy demonstrated high acceptability and low dropout rates, making it particularly suitable for adolescents with mild to moderate depression (44). In a large-scale randomized controlled trial, Rohde et al. compared the preventive effects of group cognitive behavioral therapy, bibliotherapy, and a psychoeducational pamphlet on depression. After a two-year follow-up, the incidence of new-onset depression in the bibliotherapy group was 3%, lower than in the group CBT (14%) and control (23%) groups. While the study suggests that bibliotherapy might have potential for long-term depression prevention and may be cost-effective (approximately $8 per participant), it did not show significant improvement in initial depressive symptoms. Moreover, baseline differences among groups may have influenced the outcomes (45).

However, these positive findings need to be interpreted within the broader context of existing evidence. A systematic review by Gualano et al. (46) highlighted several important limitations in this field: among the eight long-term randomized controlled trials included, all studies involving adults reported symptom reductions, whereas four trials focusing on adolescents failed to show significant effects (46). This contrast suggests that the long-term efficacy of bibliotherapy in adolescents may be unstable and could be susceptible to factors such as developmental stage, intervention format, or sample characteristics. Therefore, although preliminary results are encouraging, the application potential and underlying mechanisms of bibliotherapy in adolescent populations require further rigorous, targeted research.

### 4.2 Innovation in intervention models and diversification of formats

#### 4.2.1 Group-based bibliotherapy with structured discussion

Creative bibliotherapy has been used in mental health and social care to improve outcomes, reducing individual’s behavioral problems, enhancing social-emotional skills, and strengthening resilience and resource perception (47–51). Creative bibliotherapy programs, including community reading groups, are widely implemented in the UK as low-cost mental health interventions for vulnerable groups such as female prisoners and older adults (52, 53). Research has also explored their use in treating conditions like addiction, depression, and eating disorders (27, 54, 55).

In recent years, school- and community-based interventions have increasingly adopted group reading combined with structured discussion as a practical and scalable bibliotherapy format. This model emphasizes peer support and collaborative reflection to enhance adolescents’ abilities in emotional expression and cognitive restructuring. Although RCTs specifically evaluating this format remain limited, evidence suggests that the bibliotherapy group may be demonstrated superior long-term preventive effects on depression incidence compared to both group CBT and psychoeducational pamphlet controls, thereby potentially underscoring the added value of group-based delivery for adolescent populations (24, 56).

The structured discussion component plays a key role by creating a psychologically safe, shared space that encourages emotional disclosure and mutual understanding among participants. This feature is particularly relevant for under-resourced educational settings, such as public middle and high schools, where access to individual psychotherapy may be limited. Moreover, the relative ease of implementation and scalability makes this intervention format well-suited for widespread mental health promotion initiatives in youth populations.

#### 4.2.2 The combined application of bibliotherapy and CBT

In recent years, CBT has been widely recognized as an effective psychological intervention for treating depression. CBT achieves lasting emotional and behavioral improvements by reshaping individuals’ thoughts and beliefs (57), the therapeutic effects of creative bibliotherapy may likewise stem from encouraging readers to explore new perspectives and emotions, generating fresh insights and reinterpreting past experiences in new contexts (58). According to the National Institute for Health and Care Excellence (NICE) guidelines, bibliotherapy is recommended at Step 2 of the stepped care model for individuals with mild to moderate depressive symptoms. It is typically implemented in the form of CBT-informed self-help books and is grouped with internet-based CBT (iCBT) and group CBT as part of early psychological support strategies (59). Such interventions offer practical options for individuals facing barriers to formal care, including limited access to mental health services or reluctance to seek professional help, and thus help address the significant global treatment gap for depression (60).

Some studies have explored the integration of creative bibliotherapy with CBT (47). CBT has been shown to be effective in adolescents, outperforming both no intervention and other talk-based therapies (61, 62). In essence, CBT centers on restructuring maladaptive thinking patterns—helping individuals recognize unproductive cognitions, question their interpretations, and replace them with more balanced and realistic perspectives (63). Engagement with narratives in fiction, poetry, or film might influence mechanisms similar to those underlying CBT, fostering the development of new belief systems and perspectives among readers (64). During reading, a combination of cognitive processes—such as recognition or assimilation, and reframing or accommodation—alongside emotional responses like empathy, memory activation, and character identification, are activated (36, 65). These cognitive operations facilitate the detection of maladaptive thought patterns and the generation of more balanced interpretations. Simultaneously, emotional engagement enables individuals to confront unexamined or unhelpful beliefs, gaining new insights through immersion in fictional experiences and learning to understand others’ situations from alternative viewpoints (65). Furthermore, a meta-analysis by Richards and Richardson concluded that guided bibliotherapy—accompanied by structured support such as goal setting and task review—produces significantly better clinical outcomes compared to unguided interventions (66). These findings are consistent with earlier reviews which identified bibliotherapy as a legitimate and cost-effective method of behavioral intervention (67).

### 4.3 Integrating bibliotherapy with mindfulness and emotion regulation training in adolescent mental health interventions

The integration of bibliotherapy with mindfulness practices and emotion regulation training offers a novel approach to enhancing adolescents’ emotional awareness and regulation abilities. Research has shown that mindfulness, as an effective emotional regulation strategy, can significantly improve adolescents’ emotional states and may reduce the incidence of depression, anxiety, and related psychological issues (68). Incorporating mindfulness elements into bibliotherapy can help adolescents better recognize and accept their emotions, thereby strengthening their capacity to cope with negative experiences (69). Mindfulness emphasizes present-moment awareness and non-judgmental acceptance—an approach particularly beneficial for adolescents, who are often emotionally sensitive and vulnerable during puberty. This method helps reduce rumination and enhances self-regulation (70).

Emotion regulation training, another key intervention, typically includes modules such as emotional identification, expression, and management. These structured programs can systematically enhance adolescents’ understanding of their emotional responses and coping strategies (71). Within this framework, bibliotherapy serves as a valuable tool that uses narratives and character experiences to help adolescents better understand complex emotional states, thereby potentially fostering empathy and emotional regulation through story immersion (72). In the field of cognitive literary studies, extensive research has examined how narrative immersion—also referred to as absorption or transportation—interacts with readers’ empathic engagement. Numerous empirical studies have explored whether and how reading literary fiction contributes to the development of empathy and perspective-taking (73–75). Building on these findings, bibliotherapy utilizes narrative and character experiences to draw readers into emotionally resonant stories, fostering empathy and deepening emotional understanding. Such immersive engagement is thought to enhance perspective-taking and emotional awareness, thereby possibly contributing to emotional regulation and psychological reflection.

Moreover, the combination of bibliotherapy and mindfulness creates a psychologically safe and non-judgmental space for adolescents to explore and express their emotions without fear of external criticism or misunderstanding (72). Reading materials with mindfulness-related themes allow adolescents to observe characters facing emotional challenges and learn how to respond to their own emotions with openness and acceptance, which helps reduce fear of negative emotions (76). In practice, simple mindfulness meditation before reading can enhance concentration, clear mental distractions, and improve emotional engagement with the text (77, 78).

### 4.4 The feasibility and application of digital bibliotherapy in adolescent mental health interventions

Digital bibliotherapy has emerged as a promising mental health intervention and has garnered increasing attention in recent years, particularly among adolescents. Existing studies suggest that this format can be effective and well-accepted in addressing emotional problems such as depression and anxiety (79, 80). With the widespread adoption of digital technologies, adolescents—being a population highly engaged with mobile devices and the internet—show a high level of adaptability and acceptance toward digital interventions. This makes the promotion of digital bibliotherapy within this age group both feasible and valuable.

Research further indicates that mobile health (mHealth) applications might help overcome traditional barriers to accessing psychological services, especially in low-resource settings. For instance, in adolescent cancer patients, mobile app–based psychological interventions have shown good acceptability and preliminary effectiveness, highlighting high engagement levels with digital bibliotherapy among youth (81).

Several factors influence adolescents’ engagement with digital bibliotherapy, among which design and user experience are particularly critical. Studies show that ease of use and user-friendly interfaces significantly impact adolescents’ willingness to adopt and continue using digital interventions (82). For example, the Spark program improved user participation through intuitive interfaces and engaging interactive elements (82). Furthermore, personalization and content tailored to the developmental needs of adolescents could enhance both acceptability and user engagement.

There is a close relationship between user experience and treatment adherence. High-quality digital experiences not only attract initial interest but may also support sustained engagement over time. Research indicates that the acceptability of digital interventions is closely linked to their interactivity, enjoyment, and feedback mechanisms (83). For example, the HealthySMS system, which uses daily reminders and interactive content, was shown to improve adolescent treatment adherence by enhancing their sense of involvement (84). The flexibility in timing and location offered by digital platforms also aligns well with the lifestyle of adolescents, contributing to improved adherence.

Despite the promising feasibility of digital bibliotherapy among adolescents, further research is necessary to evaluate its effectiveness and long-term impact across diverse settings. Differences in cultural background, geographic location, and psychological conditions may influence adolescents’ acceptance of digital interventions. Therefore, more targeted studies and culturally sensitive adaptations are warranted (85, 86).

### 4.5 The potential downsides of bibliotherapy

Although bibliotherapy is widely regarded as a low-cost and easily implemented psychological intervention, its potential negative effects also warrant attention. Troscianko et al. found in the field of eating disorders that reading novels or memoirs unrelated to the illness generally had neutral or positive effects on readers’ emotions, self-esteem, body perception, and behavior, whereas narratives centered on illness experiences were often reported to have a “significant negative impact” (87). In summary, although bibliotherapy interventions generally demonstrate positive therapeutic effects, existing research indicates that under certain circumstances, they may elicit emotional triggering or other maladaptive responses (88, 89). Therefore, these potential risks should be acknowledged in research and clinical applications to maintain balance and scientific rigor in academic discussions.

## 5 Challenges and future directions

### 5.1 Limitations of existing research

Although bibliotherapy has shown promising potential in the intervention of adolescent depression, existing studies still face several limitations, including inadequate research design, short intervention periods, and weak clinical support systems. Due to the diversity of reading materials and formats, some studies suffer from small sample sizes, which limits the statistical power of their findings. Moreover, reading is a continuous and gradual process, making it difficult to accurately assess therapeutic outcomes within a fixed time frame. The effectiveness of bibliotherapy can also be influenced by multiple factors such as text selection, readers’ comprehension abilities, and cultural differences. Given the diversity of reading materials and the limited scale of current studies, future research should conduct more systematic investigations to develop targeted and generalizable models of reading-based interventions.

### 5.2 Future research directions

#### 5.2.1 Multicenter large-sample studies

Future studies should adopt multicenter, cross-regional large-sample designs to systematically evaluate the effectiveness and safety of bibliotherapy in adolescents with depressive disorders. For example, collaboration among schools, community mental health service centers, and psychiatric departments across different regions could enable the recruitment of over 1,000 adolescent participants for follow-up periods exceeding one year. A comprehensive dataset—including psychological assessments, physiological indicators, and quality of life measures—could enhance the representativeness and generalizability of the findings.

#### 5.2.2 Exploration of neuroscientific mechanisms

Neurobiological studies have shown that reading activities can activate several key brain regions involved in emotional regulation and cognitive functions, particularly the prefrontal cortex and the amygdala (90–92). In recent years, researchers have used functional magnetic resonance imaging (fMRI) to investigate the effects of reading on the adolescent brain. Findings indicate that reading not only engages the prefrontal cortex and amygdala but also modulates other regions involved in emotional and cognitive processing, such as the hippocampus and the cingulate gyrus (93, 94). However, neurobiological research specifically focused on bibliotherapy remains limited. To better understand the biological mechanisms underlying bibliotherapy, future studies should integrate neuroimaging techniques, including fMRI and resting-state connectivity analyses, to examine changes in key brain regions associated with emotional processing, cognitive control, and self-referential thinking (e.g., the amygdala, anterior cingulate cortex, and prefrontal cortex) during reading activities. Such findings would provide a neurobiological foundation for the development of more precise and effective intervention strategies.

#### 5.2.3 Development of personalized reading programs

Leveraging artificial intelligence, big data analytics, and natural language processing, intelligent personalized reading recommendation systems can be developed to enable individualized interventions. For instance, by analyzing adolescents’ psychological profiles, reading preferences, cognitive abilities, and emotional states, the system could dynamically recommend appropriate reading materials, improving engagement and compliance. Furthermore, the system could track reading behaviors and provide real-time feedback to support ongoing intervention evaluation and adjustment.

#### 5.2.4 Establishment of a professional training system

To ensure high-quality clinical implementation of bibliotherapy, a structured and standardized training system for professionals should be established. This may include the development of unified training curricula, technical manuals, and assessment standards, with training delivered through a combination of online and offline formats. Target trainees could include psychotherapists, school counselors, and social workers, ultimately building a well-qualified team capable of delivering bibliotherapy effectively and professionally. In addition, future research may draw on the systematic experience of the UK organization The Reader in training facilitators for “shared reading,” in order to develop a standardized training system for reading therapy.

#### 5.2.5 Cross-cultural adaptation studies

Cultural factors play a critical role in shaping the acceptance and efficacy of bibliotherapy. Future research should focus on validating the intervention across diverse cultural settings and exploring how dimensions such as language, values, and reading preferences influence therapeutic outcomes. For example, in Eastern cultures, emotional restraint and implicit communication may affect how adolescents resonate with reading content, whereas in Western cultures, open attitudes toward self-expression and mental health may facilitate greater acceptance. Conducting cross-cultural comparative studies will be essential for enhancing the global applicability and cultural sensitivity of bibliotherapy.

#### 5.2.6 Ethical and privacy concerns in clinical implementation

With the growing prevalence of digital reading platforms and mental health apps, the digitalization and remote delivery of bibliotherapy is becoming increasingly common. However, ethical concerns, particularly regarding personal privacy and data security, must be carefully addressed. Adolescents are particularly sensitive to the protection of their personal information, especially when it involves mental health data. Therefore, future research should explore effective mechanisms for ensuring informed consent, implementing robust data encryption, and managing information access permissions. These measures are vital for preventing data breaches or misuse and for fostering trust and willingness among adolescent participants.

#### 5.2.7 Creative integration with complementary methods

Future research should explore the creative integration of bibliotherapy with other evidence-based therapeutic approaches to develop synergistic interventions that may enhance overall efficacy. For example, combining bibliotherapy with mindfulness meditation is a promising direction; a structured session could begin with a guided meditation to help participants achieve a state of relaxed awareness and emotional receptivity, followed by the reading of selected texts to facilitate deeper cognitive-emotional engagement. Similarly, post-reading expressive writing or art-based activities could be incorporated to help individuals consolidate insights and process emotions non-verbally. Future studies should employ randomized controlled trials to rigorously compare these multimodal protocols against standard bibliotherapy, examining their additive effects on key outcomes such as emotional regulation, self-awareness, and intervention adherence. Investigating the underlying mechanisms—such as whether meditation augments the effects by reducing cognitive barriers to text immersion—will be crucial for optimizing these combined approaches.

## 6 Conclusion and outlook

As a non-pharmacological intervention, bibliotherapy demonstrates promising potential in addressing adolescent depression. By fostering emotional resonance, promoting cognitive restructuring, and providing social support, it can effectively alleviate depressive symptoms. With growing societal attention to adolescent mental health, the application prospects of bibliotherapy are becoming increasingly broad. Future research should focus on refining intervention models to enhance their scientific basis, precision, and effectiveness. Additionally, interdisciplinary collaboration is essential—integrating resources from psychology, education, and computer science to drive innovation in both the theory and practice of bibliotherapy. Through joint efforts, bibliotherapy is expected to offer more diverse and effective approaches for the prevention and treatment of adolescent depression.
